# Three New Pierce's Disease Pathogenicity Effectors Identified Using *Xylella fastidiosa* Biocontrol Strain EB92-1

**DOI:** 10.1371/journal.pone.0133796

**Published:** 2015-07-28

**Authors:** Shujian Zhang, Pranjib K. Chakrabarty, Laura A. Fleites, Patricia A. Rayside, Donald L. Hopkins, Dean W. Gabriel

**Affiliations:** 1 Plant Pathology Department, University of Florida, Gainesville, Florida, United States of America; 2 Mid-Florida Research and Education Center, University of Florida, Apopka, Florida, United States of America; University of the West of England, UNITED KINGDOM

## Abstract

*Xylella fastidiosa* (*X*. *fastidiosa*) infects a wide range of plant hosts and causes economically serious diseases, including Pierce's Disease (PD) of grapevines. *X*. *fastidiosa* biocontrol strain EB92-1 was isolated from elderberry and is infectious and persistent in grapevines but causes only very slight symptoms under ideal conditions. The draft genome of EB92-1 revealed that it appeared to be missing genes encoding 10 potential PD pathogenicity effectors found in Temecula1. Subsequent PCR and sequencing analyses confirmed that EB92-1 was missing the following predicted effectors found in Temecula1: two type II secreted enzymes, including a lipase (LipA; PD1703) and a serine protease (PD0956); two identical genes encoding proteins similar to Zonula occludens toxins (Zot; PD0915 and PD0928), and at least one relatively short, hemagglutinin-like protein (PD0986). Leaves of tobacco and citrus inoculated with cell-free, crude protein extracts of *E*. *coli* BL21(DE3) overexpressing PD1703 exhibited a hypersensitive response (HR) in less than 24 hours. When cloned into shuttle vector pBBR1MCS-5, PD1703 conferred strong secreted lipase activity to *Xanthomonas citri*, *E*. *coli* and *X*. *fastidiosa* EB92-1 in plate assays. EB92-1/PD1703 transformants also showed significantly increased disease symptoms on grapevines, characteristic of PD. Genes predicted to encode PD0928 (Zot) and a PD0986 (hemagglutinin) were also cloned into pBBR1MCS-5 and moved into EB92-1; both transformants also showed significantly increased symptoms on *V*. *vinifera* vines, characteristic of PD. Together, these results reveal that PD effectors include at least a lipase, two Zot-like toxins and a possibly redundant hemagglutinin, none of which are necessary for parasitic survival of *X*. *fastidiosa* populations in grapevines or elderberry.

## Introduction

*Xylella fastidiosa* (*X*. *fastidiosa*) is a fastidious, insect-vectored, plant xylem-limited bacterial pathogen that causes a variety of diseases in a wide range of plant species [1]. Among the most damaging *X*. *fastidiosa* diseases are Pierce’s disease (PD) of *Vitis viniferea* grapevines, leaf scorch of almonds, and citrus variegated chlorosis (CVC). In grapevines, *X*. *fastidiosa* cells multiply and spread widely from the site of infection to colonize the xylem of susceptible grapes. The symptoms of PD include leaf scorch and xylem occlusion, resulting in the eventual death of the vine [1]. Among the earliest symptoms caused by *X*. *fastidiosa* on grape is a characteristic leaf scorch that, although similar to symptoms of water stress, often occurs in bands, which is suggestive of either toxin or pathogenicity effector activity [2–4].

Genomic comparisons of various *X*. *fastidiosa* genomes to those of other bacterial pathogens have resulted in the identification of a limited number of potential virulence factors secreted through Type I, II and V secretion systems [5–7]. *X*. *fastidiosa* lacks genes encoding Type III secretion system machinery as well as Type III effectors. The Type I secretion system is involved in processes such as drug resistance via multidrug efflux as well as secretion of effectors [8]. *X*. *fastidiosa tolC*, which encodes the predicted outer membrane component of Type I secretion, was shown to be both functional and necessary for *X*. *fastidiosa* pathogenicity and *in planta* survival of *X*. *fastidiosa* in *V*. *vinifera* grapevines [9]. *X*. *fastidiosa* genomes include multiple genes encoding predicted type I effectors, including repeats in toxin (Rtx) family members, hemolysins and bacteriocins.

*X*. *fastidiosa* has also been shown to have all essential genes required for Type II secretion [5]. Proteins secreted by this system include proteases, cellulases, pectinases, phospholipases, lipases, and toxins. In general, extracellular enzymes are required for the hydrolysis of different components of the plant cell wall and contribute to cell damage and disease [10]. Lipases (EC 3.1.1.3) are lipolytic enzymes which constitute a special class of carboxylester hydrolases (EC 3.1.1) capable of releasing long-chain fatty acids from natural water-insoluble esters (lipids). Lipases typically carry a LIP domain and are secreted pathogenicity factors of both bacterial and fungal pathogens of animals and plants [11–14]. There are three putative lipases, PD1702, PD1703 and PD1211, found in the Temecula1 genome, but no reports of any potential role in pathogenicity of *X*. *fastidiosa*.

Type V secretion systems include the autotransporter pathway (type Va or AT-1), the two partner secretion pathway (type Vb), and the type Vc pathway (AT-2) [15]. *X*. *fastidiosa* proteins reported to be secreted through this secretion system include two comparatively large hemagglutinin-like proteins, PD2118 (3457 aa) and PD1792 (3377 aa), through type Vb pathway [16] and an AT-1 autotransporter (PD0528) through type Va pathway [7]. The two Type V secreted hemagglutinin-like proteins (PD1792 and PD2118) contributed to *X*. *fastidiosa* biofilm formation and colonization and were important for insect vector transmission [16–18]. Knockout mutations of either of these large hemagglutinins results in hypervirulence and greatly increased PD symptoms [16]. There are another four smaller predicted hemagglutinin-like proteins (PD0986, PD2108, PD2110 and PD2116; 376–438 aa) found in *X*. *fastidiosa* Temecula1 but at least one (PD0986) appeared missing in *X*. *fastidiosa* EB92-1 [19]. The potential role or possible functionality of these four, evidently redundant and smaller hemagglutinin proteins in *X*. *fastidiosa* pathogenicity and potential for secretion are unknown.

*X*. *fastidiosa* EB92-1 is a well characterized and effective PD biocontrol strain isolated from elderberry which can be inoculated in a single location in a grapevine and the entire plant is protected from PD for years [20]. EB92-1 causes only very slight symptoms on grapevines, even under ideal conditions, thus providing a natural platform strain to test hypotheses relating to pathogenicity effector function. The purpose of this study was to utilize this infectious but essentially nonpathogenic strain EB92-1 to attempt to identify pathogenicity effectors present in PD causing strain Temecula1 but missing from EB92-1. In this study, three of these missing genes, PD1703 (LipA), PD0928 (Zot) and PD0986 (hemagglutinin), were functionally tested and all three were found to enhance PD symptoms in EB92-1 on *V*. *vinifera* grapevines.

## Materials and Methods

### Bacterial strains, plasmids and media

All plasmids, *X*. *fastidiosa* strains, *Xanthomonas citri* B21.2 and *E*. *coli* strains used are listed in Table 1. *X*. *fastidiosa* cells were grown at 28°C in PD3 medium: tryptone (4 g/L), soytone peptone (2 g/L), trisodium citrate (1 g/L), disodium succinate (1 g/L), MgSO_4_·7H_2_O (1 g/L), K_2_HPO_4_ (1.5 g/L), KH_2_PO_4_ (1 g/L), and 0.1% (wt/vol) Hemin chloride (10 mL/L); pH was adjusted to 7.0, and soluble potato starch (2 g/L) and, optionally, agar (15 g/L) were added prior to sterilization by autoclave for 20 min [21]. *Xanthomonas* cells were routinely grown on nutrient Broth (Becton, Dickinson and Co., Sparks, MS, USA) at 28°C. *E*. *coli* strains were grown on Luria-Bertani medium at 37°C.

**Table 1:** Bacterial strains and plasmids used.

Strains and plasmids	Relevant genotype or description^a^	Source or reference^b^
***Xylella fastidiosa***		
Temecula1	Wild type	[22]
EB92-1	Biocontrol strain	[20]
***Escherichia coli***		
Mach1-T1^R^	F- φ80(*lacZ*)ΔM15 Δlac*X74* *hsdR*(r_k_^‒^m_k_^+^) Δ*recA*1398 *endA1 tonA*	Invitrogen
BL21(DE3)	F–*omp*T *hsd*SB(rB–, mB–) *gal dcm* (DE3)	Invitrogen
***Xanthomonas***		
B21.2	X.*citri* 3213^T^ (*pthA*::Tn*5*-*gusA*), Sp^r^Km^r^Tc^r^, marker-exchanged mutant	[23]
**Plasmids**		
pBBR1MCS-5	Rep *Bordetella*, *lac*Z, Gm^r^	[24]
pGEM-T	Ap^r^, pUC ori, *lac*Z	Promega Corp.
pET-27b(+)	Km, f1 and pBR322 ori,	EMD Millipore
pSZ24	pGEM-T with 1982 bp PCR product of PD1703 including native promoter region, Ap^r^	This study
pSZ25	pGEM-T with 2286 bp PCR product of PD1702 including native promoter region, Ap^r^	This study
pSZ26	pBBR1MCS-5 with *Apa*I/*Cla*I fragment from pSZ24, Gm^r^	This study
pSZ28	pBBR1MCS-5 with *Apa*I/*Cla*I fragment from pSZ25, Gm^r^	
pSZ40	pGEM-T with 1446 bp PCR product of PD0928, including native promoter region, Ap^r^	This study
pSZ41	pBBR1MCS-5 with *Bam*HI/*Sal*I fragment from pSZ40, Gm^r^	This study
pPC2.5	pGEM-T with 1194 bp PCR product of PD0986, including native promoter region, Ap^r^	This study
pPC3.1	pBBR1MCS-5 wtih *EcoR*I/ *Bam*HI fragment from pSZ40, Gm^r^	This study
pSZ35	pGEM-T with 1170 bp PCR product of revised PD1703 (*lipA*), without secretion signal. Ap^r^	This study
pSZ37	pET-27b(+) with *Msc*I/*Nhe*I fragment from pSZ35. Km^r^	This study

^a^ Gmr, Apr, and Kmr = gentamycin, ampicillin, and kanamycin resistant, respectively; PCR = polymerase chain reaction.

^b^ Sources: Invitrogen Life Technologies, Carlsbad, CA, USA, Promega Corporation, Madison, WI, USA, EMD Millipore, Billerica, MA, USA,

### Molecular and bioinformatics techniques

DNA primers used were synthesized by Integrated DNA Technologies (IDT, Coralville, IA, USA). Total DNA was extracted from Temecula1 and EB92-1 cells using the GenElute Bacterial Genomic DNA Kit (Sigma, St. Louis, MO, USA). Plasmid DNA was extracted from *E*. *coli* using the QIAGEN (Valencia, CA, USA) QIAprep Spin Miniprep kit. PCR purification and gel extraction were done using the QIAGEN QIAquick PCR purification and Gel Extraction kits. DNA fragment ligations were performed using pGEM-T Vector Systems (Promega Corp, Madison, WI, USA). Protein BLAST (Basic Local Alignment Search Tools; BLASTP) was used to search bacterial protein sequence databases (http://blast.ncbi.nlm.nih.gov). Secretion signals were predicted using SignalP 3.0 (http://www.cbs.dtu.dk/services/SignalP). Predictions of open reading frames (ORF) were conducted using GeneMark (http://exon.gatech.edu/GeneMark/). The phylogenetic tree was generated using AlignX, Vector NTI Advance 10 (Invitrogen, Carlsbad, CA, USA) with the default set of multiple alignment parameters.

### Construction of shuttle and expression vectors

Primers used for PCR amplification of the target genes in this study are listed in Table 2. For Revised PD1703, a 1982 bp PCR fragment was amplified from Temecula1 genomic DNA using primers PDLPwp-F and PDLP03-R to amplify the region covering the PD1703 corrected putative ORF (including a predicted secretion signal leader sequence missing from the original annotation) and including its 691 bp native promoter region. This fragment was cloned into pGEM-T, resulting in pSZ24. This construct was verified by sequencing and the entire gene plus promoter was recloned using *Apa*I and *Cla*I into pBBR1MCS-5 (downstream from the *lacZ* promoter), to make pSZ26. Similarly, the constructs containing the native promoters and the ORF regions of revised PD1702, PD0928 and PD0986 were cloned into pGEM-T to form pSZ24, pSZ40 and pPC2.5, respectively (Table 1). The inserts from these three constructs were recloned into pBBR1MCS-5 using appropriate enzymes as listed in Table 1, resulting in pSZ28, pSZ41 and pPC3.1 respectively. For protein expression of revised PD1703 in *E*. *coli* BL21(DE3), the corrected putative ORF was amplified using primers PD1703-AF and PD1703-AR and cloned into pGEM-T without its predicted native secretion signal to form pSZ35. The sequence-verified insert was cloned into pET-27b (+) to create a translational fusion with the *pelB* leader to form pSZ37.

**Table 2:** Primers used in this study.

Primer name	Sequence (5’-3’)	Gene target
PDLPwp-F	GGGCCCTGCCGCATTTGAGGCTGGC	PD1703
PDLP03-R	ATCGATACGTTTGCAAACCCTATATCC	
PDLP02wp-F	GGGCCCGGATATAGGGTTTGCAAACGT	PD1702
PDLP-R	ATCGATAGGGTACATTTACCAGACCG	
ZOT-F	GTCGACCTGGTTGGGCATTAATTGGG	PD0928
ZOT-R	GGATCCTCATGGAAGCGACCCCGC	
PD0986-EcoRI-F2	CGGAATTCAGGAAGCAGTGACGGGCTG	PD0986
PD0986-BamHI-R	CGGGATCCTTAGTAGGGGGTGAGGGACT	
PD1703-AF	TGGCCAGCCGTGGGATGGTGATTAACAGA	PD1703
PD1703-AR	GCTAGC CTAACGTTTCTTCTCTAGAAAC	
RST31	GCGTTAATTTTCGAAGTGATTCGATTGC	*X*. *fastidiosa* marker gene [25]
RST33	CACCATTCGTATCCCGGTG	

### Preparation and Electroporation of *X*. *fastidiosa* and *X citri* cells

*X*. *fastidiosa* cells were grown on PD3 agar plates at 28°C. *X*. *fastidiosa* liquid cultures were grown in liquid PD3 with rotary shaking at 28°C at 120 rpm. To make *X*. *fastidiosa* cells competent for electroporation, 1 mL of 5 day old liquid *X*. *fastidiosa* culture was transferred into 35 mL of fresh PD3 broth and grown until the OD_600_ of the culture reached ~0.1. These liquid cultures (36 mL) were transferred to flasks containing 250 mL of PD3 broth and grown to OD_600_ = 0.1. The cell suspensions were chilled on ice for 30 min and cells recovered by centrifugation at 4°C for 15 min at 4,000 rpm. Cells were washed successively in 250 mL, then in 125 mL ice-cold sterile distilled water and then in 10 mL of ice-cold 10% glycerol; each wash was followed by centrifugation and resuspension by gentle hand shaking or tapping. Finally, the cells were resuspended in 1 mL of ice-cold 10% glycerol. Forty μL aliquots of concentrated cells were placed in 1.5 ml sterile tubes kept at -20°C before use and the aliquots were frozen in liquid nitrogen and either used immediately for electroporation or stored at -80°C for later use. Plasmids used for electroporation were treated with CpG Methyltransferase (New England Biolabs, Ipswich, MA, USA) [26]. One μL TypeOne Restriction Inhibitor (EPICENTRE, Madison, WI, USA) and less than 5 μL of 0.5 μg~1 μg treated plasmid DNA was added to competent cells thawed and kept on ice and mixed by pipetting. The *X*. *fastidiosa* cell-DNA mixture was transferred into 1mm electroporation cuvettes (pre-chilled on ice for 20 min before use) and electroporated at 1800 Volts (time constant usually 4.8~6.2 ms). One mL of PD3 broth without antibiotics was added into cuvette and mixed quickly by pipetting, then all cultures were transferred into 5 mL sterile tubes, sealed with parafilm and shaken at 120 rpm, 28°C for 24hrs. Cultured cells were spread (100 μL per plate) on solid PD3 medium with gentamycin at10 μg/mL. Plates were incubated at 28°C for 7–12 days and single colonies were picked for PCR analysis. *X*. *citri* B21.2 electrocompetent cells were prepared and electroporation conducted as described by Swarup et al [23].

### Lipase indicator assays on plates

An *in vitro* lipase assay was conducted using Tween 20 as the substrate and 0.01% Victoria Blue B as indicator, essentially as described by Samad et al [27]. Agar plates containing the substrate and indicator were poured and wells were created by removal of agar with a sterile cork borer (0.7 cm diam.). For native secreted lipase assays, 50 μL supernatants from centrifuged *E*. *coli*, *X*. *fastidiosa* and *X*. *citri* cell cultures grown to late mid-log phase (OD_600_ = 0.7) were added to the wells and the plates were incubated overnight at 37°C for *E*. *coli* and 28°C for *X*. *fastidiosa* and *X*. *citri* supernatants. For recombinant, over-expressed lipase assays, crude protein was extracted from *E*. *coli* BL2 (DE3) carrying pET27b and expressing PD1703 using a QIAGEN (Valencia, CA, USA) Ni-NTA Spin Kit. Protein was resuspended in 200 μL of supplied buffer and quantified with a NanoDrop 2000 spectrophotometer (NanoDropTechnologies, Wilmington, DE, USA); some protein remained insoluble but was applied as a suspension and labeled "total protein". To recover soluble protein, total protein was centrifuged at 10,000 X g for 30 min at 4°C to pellet the insoluble protein, and the supernatant was used and labeled as "supernatant". Both 50 μL of total and 50 μL of supernatant protein were tested in the agar plate assays for recombinant, over-expressed lipase.

### Hypersensitive reaction (HR) assays of crude protein extracts

*Nicotiana tabacum* cv. SR1 (also called Petite Havana) plants were maintained at 28°C with a 12-h photoperiod. At 4 to 5 weeks after germination from seeds, three nearly fully expanded upper leaves were pressure infiltrated through the stomata using the blunt end of a 1 ml tuberculin syringe gently pressed against the abaxial leaf surface. Approximately 10 μL of crude protein extracts from *E*. *coli* BL21 (DE3) carrying empty vector (pET-27b) or expressing revised PD1703 (pET-27b::*lipA*, pSZ37) were infiltrated into a zone of about 1.5 cm diameter. Similarly, three nearly fully expanded leaves of ca. 2' tall citrus trees in pots (*Citrus sinensis* L. Osbeck cv. Hamlin) were infiltrated with 10 μL of the same crude protein extracts. All the inoculation zones on inoculated tobacco or citrus plants were circled using a black marker to indicate the extent of infiltration.

### *X*. *fastidiosa* pathogenicity assays on grapevines

For *X*. *fastidiosa* pathogenicity assays, grape (*Vitis vinifera*) cv. Carignane plants were inoculated by needle puncture as described [9]. In brief, 10μL droplets of *X*. *fastidiosa* bacterial suspensions (OD_600_ = 0.25) in SCP buffer (trisodium citrate, 1 g/L; disodium succinate, 1 g/L; MgSO_4_·7H2O, 1 g/L; K_2_HPO_4_, 1.5 g/L; and KH_4_PO_4_, 1 g/L; pH 7.0) were applied on opposite sides of each of 4–5 internodes of ca. 3 ft high grapevines in pots, starting with the second internode from the base. A sterile, tuberculin needle was used to puncture the stems to a depth of 1 to 3 mm through each of the droplets. This resulted in the droplets of suspension being drawn into the stem xylem. Plants were not watered for at least 36 hours prior to inoculation. Each experiment was conducted three times with 4–12 replications in each experiment. Inoculated plants were maintained in an air conditioned green house and carefully observed for the appearance of symptoms. Observations were recorded from the time the first visible symptoms appeared (ca. 4–6 weeks post inoculation) and continued for another 2 months. Disease severity was quantified and expressed as a % of diseased leaves (including bare petioles and bare nodes) on each inoculated plant by measuring the number of symptomatic leaves, the number of bare petioles and the number of bare nodes on each plant, and dividing by the total number of leaves (asymptomatic or symptomatic), bare petioles and bare nodes per plant.

## Results

### PCR and sequence evidence of 5 pathogenicity genes missing in EB92-1

Suspected pathogenicity genes found in Temecula1 that appeared to be missing from EB92-1 [19] were further investigated by designing PCR primers internal to and outside of the coding sequences (CDSs) of PD1703 (LipA), PD0956 (Serine protease), PD0915 (Zot), PD0928 (Zot) and PD0986 (hemagglutinin-like) (S1 Table). Two sets of primers were designed to attempt detect these genes in EB92-1; one set was designed internal to the CDS of each gene (92% of predicted EB92-1 proteins had more than 99% identity with Temecula1 proteins [19]) and at least one additional set was designed external to the CDS in Temecula1. PCR amplification results using DNA extracted from both Temecula1 and EB92-1 are shown (S1 Fig). In the cases of PD1703 and PD0956, the regions outside of the apparently missing genes were readily found based on gaps predicted by Mauve 2.3.1 within the existing EB92-1 contigs deposited in Genbank (Accession # AFDJ00000000.1). The regions of EB92-1 amplified by PCR primer sets PDLPwp-F / PDLP-R and XFEB114-F / XFEB114-R1 were sequenced to confirm that PD1703 and PD0956, respectively, were truly missing, at least from their locations in Temecula1 (additional sequence data not shown). Primers internal to the coding regions of PD1703 and PD0956 failed to amplify any products from EB92-1.

In the cases of PD0915 and PD0928, which are 100% identical in coding sequence and appear in nearly identical, 4.4 kb tandem direct repeats spaced ca. 4 kb apart in Temecula1, primer sets internal and external to both genes failed to amplify any products from EB92-1. Indeed, the entire 12.7 kb region containing genes PD0911–PD0929 appeared missing in EB92-1 (some data not shown). Similarly, in the case of PD0986, both the internal primer set and the external (2.0 kb) primer set failed to amplify; both flanking genes PD0985 and PD0987 appeared rearranged based on strong multiple BLASTn hits (3 contigs each) in EB92-1 (some data not shown).

### PD1703 ORF analysis and re-annotation

**Fig 1:**
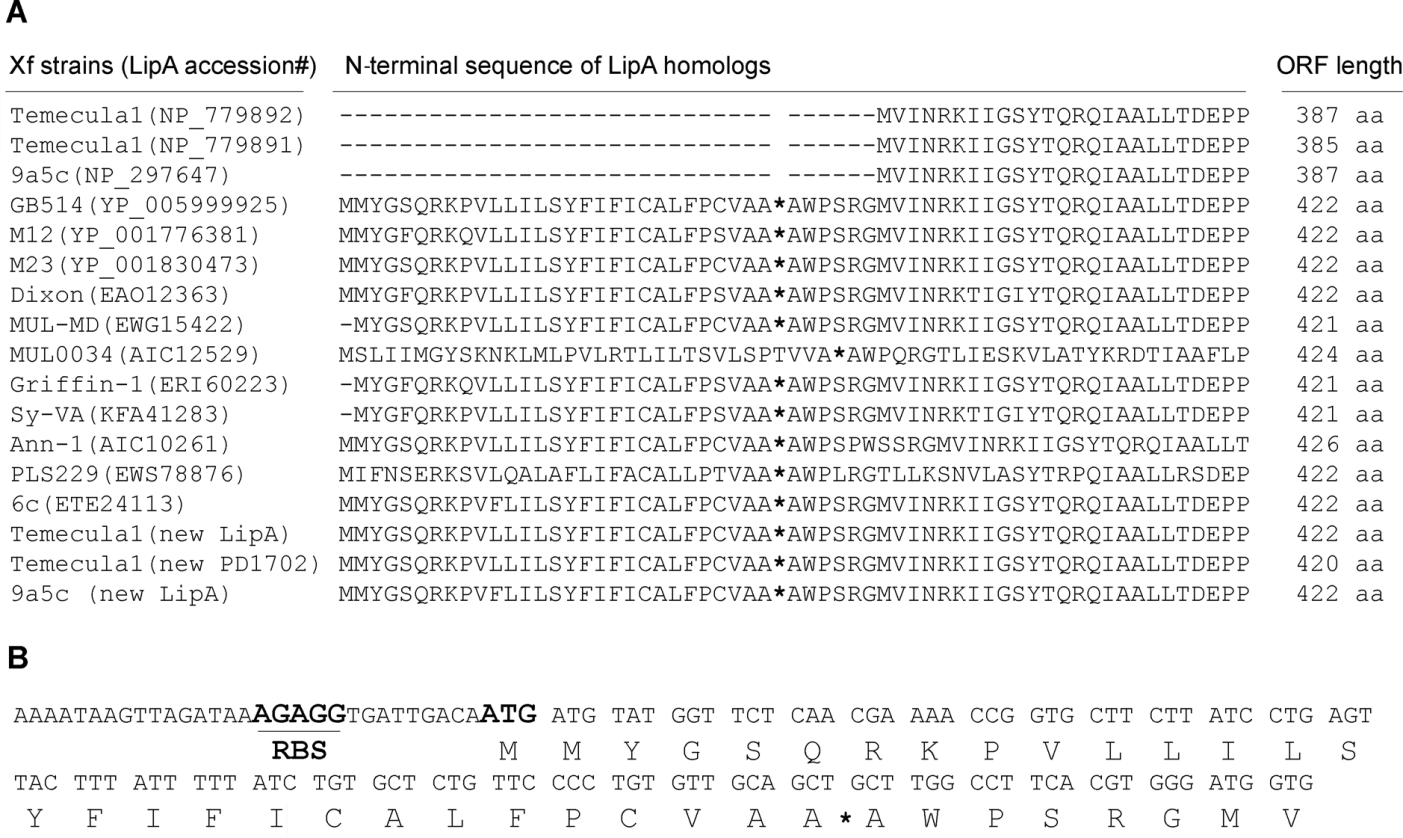
The N-terminal regions of LipA proteins from *X*. *fastidiosa*. (A) Comparative sequences of 13 *X*. *fastidiosa* strains, with accession numbers, including citrus CVC strain 9a5c, PD strain GB514, almond leaf scorch strains M12, M23 and Dixon, mulberry leaf scorch strains MUL-MD and MUL0034, oak leaf scorch strain Griffin-1, sycamore leaf scorch strain Sy-VA, oleander leaf scorch strain Ann-l, pear leaf scorch strain PLS229 and coffee leaf scorch strain 6c, as well as three re-annotations (two from Temecula1 and one from CVC). The most likely signal peptide cleavage sites for each protein are indicated by asterisks. (B) Portion of the DNA sequence including 5’ untranslated region of revised PD1703. The putative RBS and the first start codon are indicated using bold and enlarged fonts.

Protein BLAST (Basic Local Alignment Search Tools) revealed that the *X*. *fastidiosa* Temecula1 PD1703 product, which is missing from *X*. *fastidiosa* EB92-1, matched many proteins from various bacterial species annotated as secreted lipases and possessing a LIP domain (Pfam03583), including PD1702 of Temecula1 (Fig 1). The putative open reading frame (ORF) of PD1703 (GenBank accession no: NP_779892), located at nucleic acid positions 1,983,742 to 1,984,905 in the Temecula1 genome (GenBank accession no: NC_0045560) [5], encodes a predicted 387-amino-acid-long protein. The DNA sequence (nucleic acid positions 1,983,742 to 1,985,701) from *X*. *fastidiosa* Temecula1, which is annotated to contain the 3’ end of PD1704, was predicted using GeneMark to more likely belong to PD1703. This predicted LipA protein [labeled as “Temecula1 (new LipA)”, Fig 1A] is encoded by a putative ORF located in Temecula1 at nucleotides 1,983,742 to 1,985,010, with a putative ribosome biding site (RBS) “AGAGG” located 9 base pairs upstream of the ATG translational start codon. This revised LipA ORF encodes a 422-amino-acid-long protein and possesses a LIP domain (Pfam03583) between amino acid positions 132 and 203 (data not shown). Its Type II secretion signal was predicted using SignalP 3.0 with a probability of 0.993 with a likely cleavage site between positions 30^th^ and 31^st^ amino acids, entirely consistent with the other *X*. *fastidiosa* LipA homologs (Fig 1B). Similarly, Temecula1 PD1702 and XF0357 from *X*. *fastidiosa* 9a5c were found to likely be similarly mis-annotated. The N-terminal region of these revised predicted ORFs (labeled as "new PD1702" and "new LipA" are shown in Fig 1A). The revised predicted ORFs of PD1703 and PD1702 were used for further studies and functional verification in this work.

### Revised PD1703, but not PD1702, exhibited functional, secreted lipase activity in *X*. *citri*, *E*. *coli* and *X*. *fastidiosa* EB92-1

**Fig 2:**
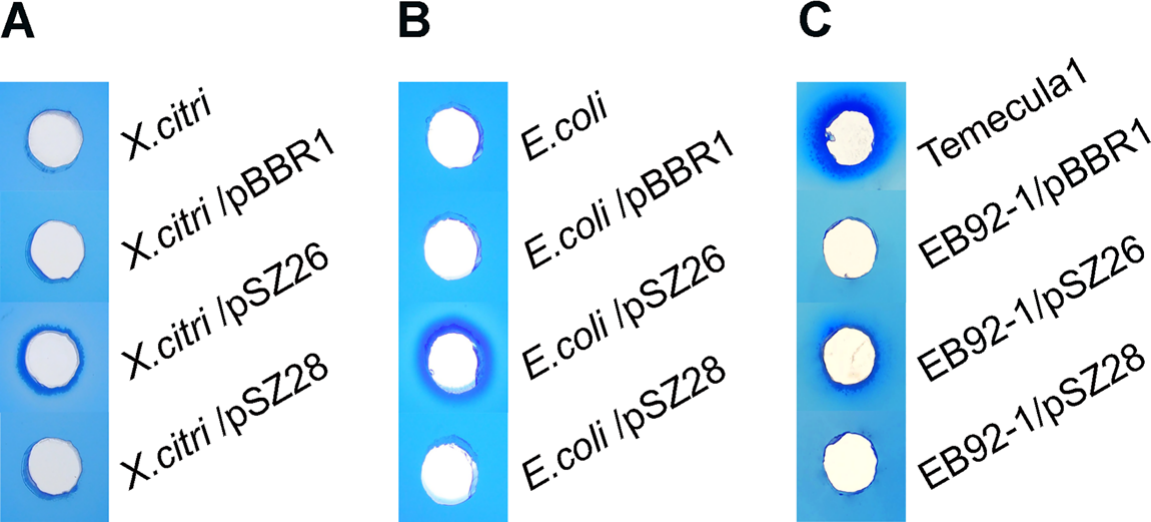
Secreted lipase activity assays of culture supernatants from *X*. *citri* B21.2, *E*. *coli* Mach1-T1R and *X*. *fastidiosa* strains Temecula1 and EB92-1. (A) Lipase assay of culture supernatants from untransformed *X*. *citri* B21.2 and transformants with empty vector pBBR1MCS-5 (pBBR1), revised PD1703 (pSZ26) and with revised PD1702 (pSZ28). (B) Lipase assay of culture supernatants from untransformed E. coli Mach1-T1R strains and transformants. (C) Lipase assay of culture supernatants from untransformed *X*. *fastidiosa* Temecula1 and EB92-1 transformants. Photos were taken at 24 hours after plating. The results shown are representative of data from three independent replicates.

The revised putative PD1703 ORF with its native promoter region (691 bp) was cloned into pBBR1MCS-5 (and downstream from the *lacZ* promoter). Similarly, the revised putative PD1702 ORF with its native promoter region (1024 bp) was cloned into pBBR1MCS-5. Both constructs were used to transform *X*. *citri* B21.2, *E*. *coli* Mach1-T1^R^ and *X*. *fastidiosa* EB92-1. The crude, cell free culture supernatants were assayed for lipase activity, without purification or concentration. As shown in Fig 2, only culture supernatants from cells carrying the revised PD1703, but not the revised PD1702, exhibited lipase activity, and in all three assayed strains (*X*. *fastidiosa*, *E*. *coli* and *X*. *citri*). Other than these, only culture supernatant from the positive control *X*. *fastidiosa* Temecula1 exhibited lipase activity (Fig 2C). Culture supernatants from the same strains or the same strains carrying the empty vector pBBR1MCS-5 (labeled as “pBBR1”) or PD1702 (labeled as "pSZ28") were all negative for lipase activity (Fig 2A, 2B and 2C).

### Crude protein extracts of revised PD1703 overexpressed in *E*. *coli* exhibited lipase activity and induced HR in tobacco and citrus

**Fig 3:**
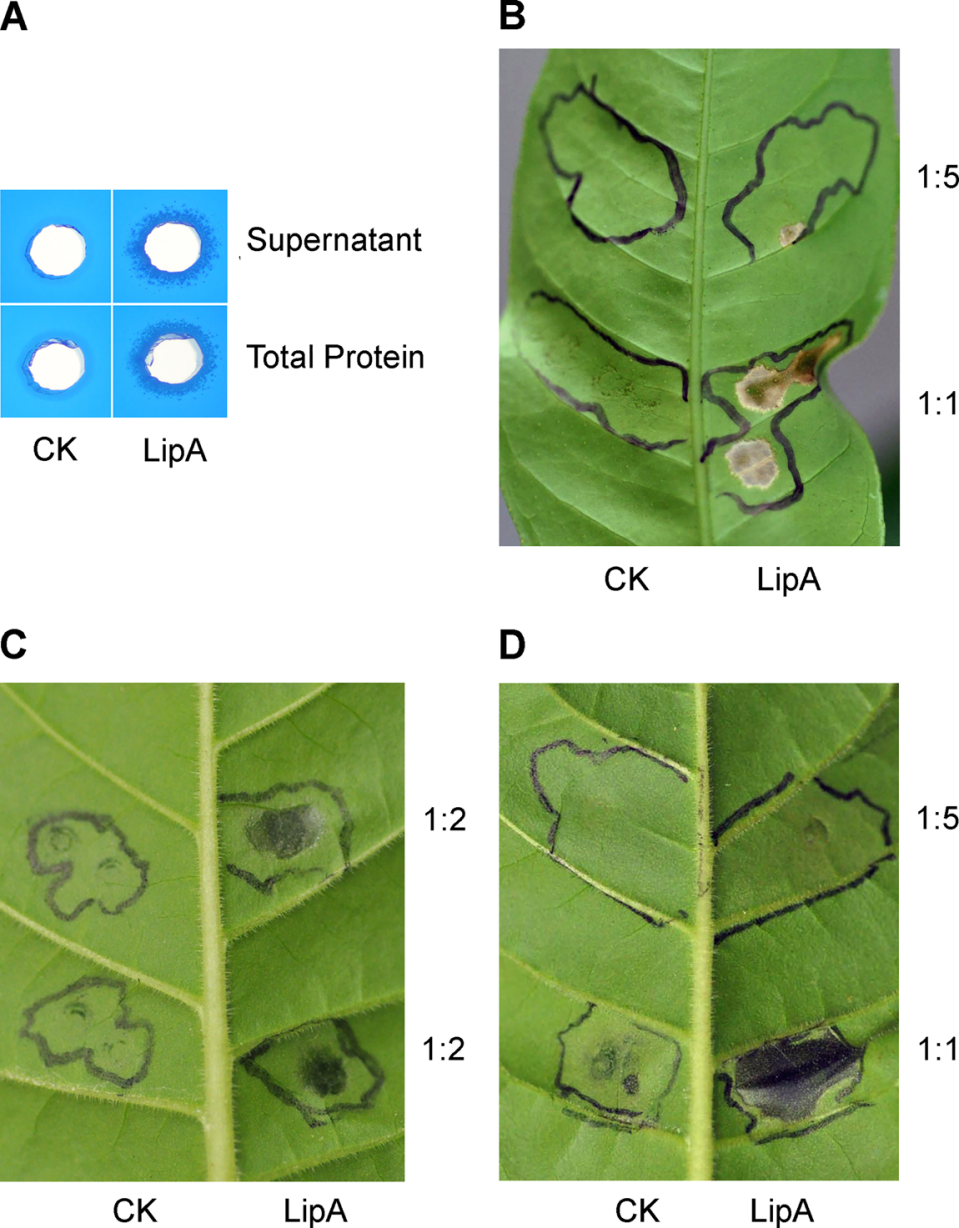
Lipase activity and plant response assays using crude protein extracts from revised PD1703 (LipA) overexpressed in *E*. *coli*. (A) Lipase activity assays of PD1703 recombinant crude protein extracts from *E*. *coli* BL21 (DE3) strains. Fifty μL of crude protein from *E*. *coli* BL21 (DE3) with empty vector pET-27b (labeled as “CK”, 36 mg/mL) or expressing pSZ37 (labeled as "Total Protein", 30 mg/mL) and applied as a suspension or clarified by centrifugation ("Supernatant") were applied in the agar assay wells. Photos were taken at 24 hours post plating. (B) Ten μL of undiluted crude protein (1:1) and 1:5 diluted extracts were inoculated into citrus leaves. Zones of inoculation were marked with black ink and the photo was taken 48 hours later (48 hpi). (C) Ten μL of 1:2 diluted crude protein extract (15–18 mg/mL total protein) was inoculated into tobacco leaves. Zones of inoculation were marked with black ink and the photo was taken 17 hours later (17 hpi). (D) Ten μL of undiluted crude protein (1:1) and 1:5 diluted extracts were inoculated into tobacco leaves. Zones of inoculation were marked with black ink and the photo was taken 48 hours later (48 hpi). Similar results were obtained in three independent experiments.

Crude protein was extracted from induced *E*. *coli* BL2 (DE3) cultures carrying pSZ37 (revised PD1703, LipA) and from empty pET27b vector control cells. SDS-PAGE gel analysis indicated that the LipA protein was induced, and much of it was insoluble (S2 Fig). Both "Total Protein" (30 mg/mL) extracts from *E*. *coli*/pSZ37 cultures expressing LipA as well as centrifuged, clarified supernatants ("Supernatants") from these extracts exhibited lipase activity in these assays, whereas slightly higher levels of "Total Protein" (36 mg/mL) extracted from the empty vector pET27b did not (Fig 3A).

Ten μL of both "Total Protein" (30 mg/mL) and clarified supernatant extracts from *E*. *coli* pSZ37 ("LipA") cultures were injected into both citrus and tobacco leaves, along with total protein (36 mg/mL) and clarified supernatant extracts from *E*. *coli* empty vector ("CK") control cultures (refer Fig 3B, 3C and 3D). Both undiluted and 1:2 dilutions of the LipA extracts elicited a rapid HR that was visible at 14 hrs post inoculation (hpi) in tobacco (Fig 3C and 3D). Similarly, undiluted (1:1) LipA protein extracts inoculated into citrus (Fig 3B) also elicited a rapid HR that was visible at 14 hpi. A 1:5 dilution of crude LipA protein that was inoculated into both hosts caused a very slight necrotic response that was visible at 48 hpi.

### Revised PD1703 (LipA), PD0928 (Zot), and PD0986 (hemagglutinin) all enhanced pathogenicity of EB92-1 on grapes

Revised PD1703 (LipA), PD0928 (Zot), and PD0986 (short hemagglutinin) were cloned with their native promoters in pBBR1MCS-5 (downstream from the *lacZ* promoter) and transformed into EB92-1 cells. EB92-1 transformants, including empty vector as a negative control, as well as Temecula1 as a positive control, were inoculated into *V*. *vinifera* Carignane grapevines. Visible symptoms of PD began to appear on plants inoculated with Temecula1 by 4–5 weeks post inoculation, and continued to develop up to 12 weeks. Most of the Temecula1 inoculated plants were killed by 12 weeks.

**Fig 4:**
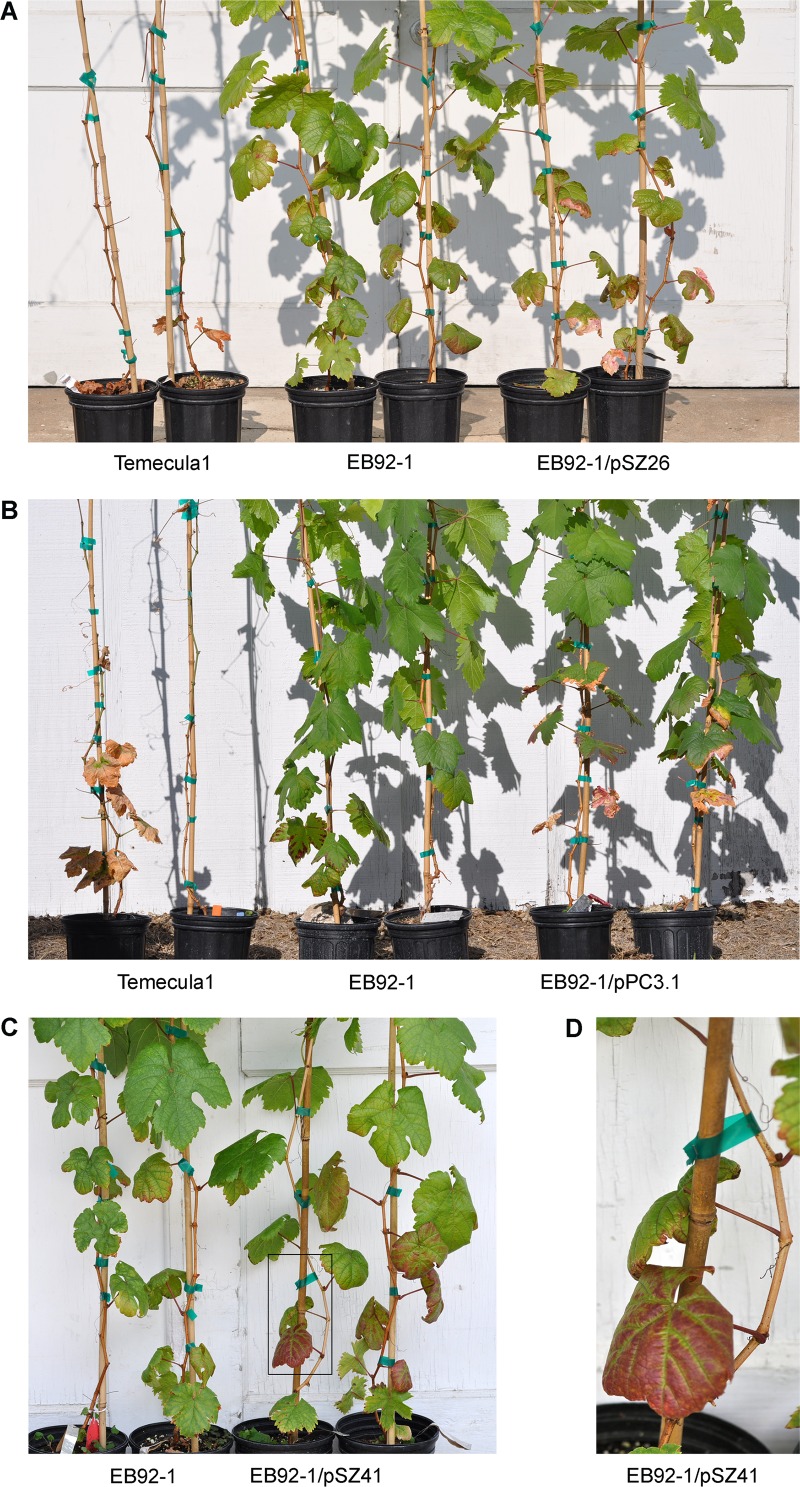
Pathogenic symptoms elicited by Temecula1, EB92-1 transformants and EB92-1 empty vector control on Carignane grapevines. (A) Comparisons of plants inoculated with Temecula1, EB92-1 (EB92-1/pBBR1MCS-5 empty vector) and EB92-1/pSZ26 (revised PD1703; LipA). (B) Comparisons of plants inoculated with Temecula1, EB92-1 and EB92-1/pPC3.1 (PD0986; hemagglutinin). (C) Comparisons of plants inoculated with EB92-1 and EB92-1/pSZ41 (PD0928; Zot). (D) Enlarged region from boxed area in (C) illustrating unique symptoms; these lower leaves abscised 10 days later. All photos were taken 90 days post-inoculation.

**Fig 5:**
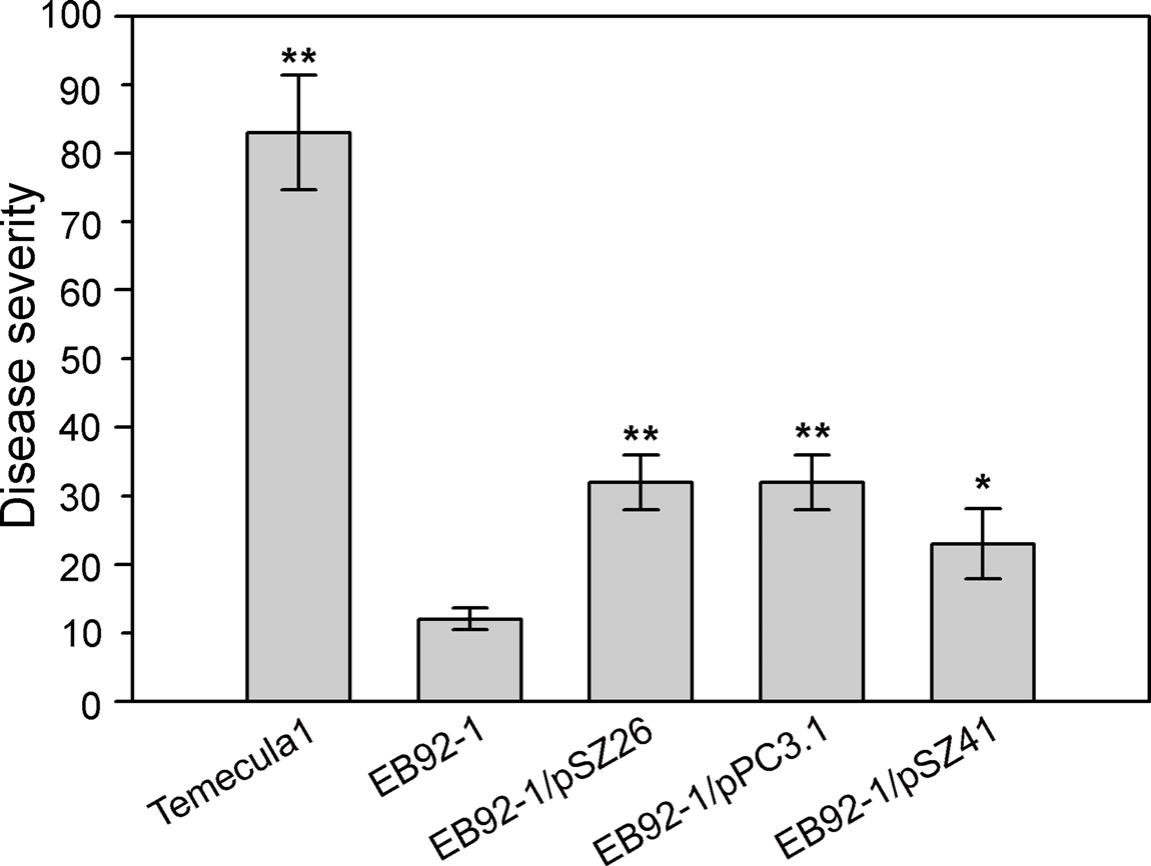
Combined disease severity data of Temecula1, EB92-1 transformants and EB92-1 empty vector control on Carignane grapevines. Temecula1, wild type PD strain; EB92-1, biocontrol strain EB92-1/pBBR1MCS-5 (empty vector); EB92-1/pSZ26, EB92-1/pSZ26 (revised PD1703, LipA); EB92-1/pPC3.1, EB92-1/pPC3.1 (PD0986, hemagglutinin), and EB92-1/pSZ41 EB92-1/pSZ41 (PD0928, Zot). Disease severity was calculated using combined data from 3 independent experiments with 4–12 replications of each inoculated strain in each experiment. Error bars indicate the standard deviations. Values marked with one or two asterisks are significantly different at the P < 0.05 and P<0.01 levels, respectively, using Student’s two-tailed *t* test.

Plants inoculated with EB92-1/pSZ26 (LipA; refer Fig 4A) showed slightly delayed pathogenesis compared with Temecula1, and visible symptoms become evident by the end of six weeks. EB92-1/pSZ26 elicited typical leaf necrosis delimited with pinkish areas that were identical in appearance to plants inoculated with Temecula1. As the infection progressed the entire lamina underwent necrosis leading to defoliation (evident in Fig 4A with Temecula1). However, infection by EB92-1/pSZ26 progressed more slowly when compared to plants inoculated with Temecula1, the symptoms remained restricted to 9–10 internodes above the lowest point of inoculation, and disease severity (ca. 32%) was greatly reduced compared with Temecula1, where infections advanced to the tip of the vine and resulted in disease severity of 83% or more. Nevertheless, inoculations using EB92-1/pSZ26 resulted in disease symptoms affecting ca. 32% of the leaves, which was significantly higher (Student *t*-test, P<0.01) than the 12% levels observed with EB92-1 (EB92-1/pBBR1MCS-5 empty vector; Fig 5).

Plants inoculated with EB92-1/pPC3.1 (hemagglutinin; refer Fig 4B) exhibited typical symptoms almost as rapidly as the wild type Temecula1 (by 5 weeks; slower than observed with EB92-1/pSZ26) but with a much slower rate of progression and affecting only ca. 32% of the leaves, as with EB92-1/pSZ26 (Fig 5). Again, these severity levels were significantly higher than the 12% levels observed with EB92-1 (P< 0.01).

Plants inoculated with EB92-1/pSZ41 (Zot; refer Fig 4C and 4D) exhibited unique, reduced visible symptoms that developed even more slowly than EB92-1/pSZ26 or EB92-1/pPC3.1, with visible symptoms becoming evident only by the end of 6 weeks. Instead of the initial necrotic symptoms typical of PD, the lower leaves (closest to the inoculation zones) of inoculated plants showed prominent anthocyanosis with green veinal areas (Fig 4C and 4D). Only as the infection advanced did the symptomatic leaves become necrotic, eventually resulting in limited defoliation and the disease did not progress very far past the inoculation zones. Nevertheless, the disease severity levels (ca 23%) were significantly higher (P< 0.05) than the 12% observed with EB92-1.

## Discussion

EB92-1 is a biocontrol strain with a genome that is highly syntenic, and nearly identical in sequence, with pathogenic Temecula1 and is notably missing multiple potential pathogenicity factors found in Temecula1 [19]. Among the missing potential pathogenicity factors is PD1703, which was predicted to encode a lipase similar to LipA of *X*. *oryzae* pv. *oryzae* (*X*. *oryzae*.). *X*. *oryzae* LipA was reported to contribute to the virulence of *X*. *oryzae* on rice by synergistically enhancing rice cell wall xylan degradation in concert with *X*. *oryzae* xylanases [13–14, 28, 29]. Purified LipA induced innate defense responses in rice, including callose deposition and HR elicitation [14].

Bacterial lipases usually contain typical N-terminal signal peptides and are exported from the bacterial cell via a type II secretion system. Alignment of the predicted protein sequences of PD1703 and PD1702 from Temecula1 and > 90% identical protein sequences from several other sequenced *X*. *fastidiosa* strains revealed that PD1702, PD1703 and XF0357 from *X*. *fastidiosa* 9a5c were missing the first 35 amino acids from the annotation, including the Type II secretion signal sequence, predicted to be present in the other *X*. *fastidiosa* annotated LipA lipases (Fig 1). The revised *X*. *fastidiosa* Temecula1 PD1703 was cloned and demonstrably functional as a lipase when overexpressed in *E*. *coli* (Fig 3); revised PD1703 significantly enhanced both PD symptoms and disease severity elicited by transformed EB92-1 cells in inoculated grapevines (Figs 4A and 5).

*X*. *fastidiosa* is a rod-shaped bacterium with dimensions ranging from 250 to 500 X 1,000 to 4,000 nm [30] and requires cell wall degrading enzymes to enable intervessel migration, as the pore sizes of intact pit membranes of grape are too small to allow passive movement of the bacteria [31]. A polygalacturonase (*pglA*) was shown to be required for the colonization and pathogenicity of *X*. *fastidiosa* on grapevines [32] and later was proved to enlarge the pore size of intervessel pit membranes with endo-1, 4-b-glucanase in a synergistic manner [33]. Based on the observed hypersensitive response on nonhosts (Fig 3), and by analogy with LipA of Xoo, revised PD1703 likely contributes to disease symptoms by helping degrade cell walls and thereby generating endogenous elicitors, which elicit an HR on nonhosts (Fig 3).

PD1703 is negatively regulated in *X*. *fastidiosa* by *gacA* [34], encoding a transcriptional regulator of genes involved in quorum sensing, toxin production, motility, biofilm formation, and extracellular polysaccharide production in many plant bacterial pathogens [35–37]. Regulation of LipA, along with other degradative enzymes, may be required to constrain inter-vessel movement by *X*. *fastidiosa* strain Temecula1. Indeed, EB92-1 spreads more slowly and titers are 10–100 fold lower than Temecula1 in grapevine [38] and some unpublished data; we speculate that absence of LipA in EB92-1 may constrain movement as well as pathogenicity of the biocontrol strain.

In addition to PD1703, two hypothetical unknown proteins are present in both Temecula1 and EB92-1 with the potential to be lipases. PD1702 and PD1211 both have predicted LIP domains, including the canonical catalytic triad residues Ser-176, His-377, and Asp-336 of *X*. *oryzae* LipA [28] (S3 Fig). Although PD1702 is 91% identical to the protein sequence of PD1703, neither EB92-1 nor any transformants of *E*. *coli* or *X*. *citri* carrying PD1702 exhibited secreted lipase activity (using Tween-20 as substrate) from culture supernatants (S2 Fig). Since the native promoters of both PD1702 and PD1703 were used, both have predicted secretion signal peptides and both were sequenced prior to recloning into wide host range shuttle vector pBBR1MCS-5, these results indicate several possibilities: 1) PD1702 may be nonfunctional as a lipase capable of degrading Tween-20; 2) the PD1702 promoter is poorly functional in EB92-1, *E*. *coli* and *X*. *citri*; 3) PD1702 is poorly secreted from EB92-1, *E*. *coli* and *X*. *citri*, or 4) an unrecognized recloning artifact resulted in loss of function. Since the recloning methodology was standard restriction digestion followed by ligation and used for both PD1702 and 1703 and since PD1703 was well secreted from EB92-1, we favor the first possibility.

PD1211 is 61% identical to the protein sequence of PD1703, and EB92-1 carries a gene 100% identical to PD1211. Nevertheless, EB92-1 did not exhibit any detected lipase activity using Tween-20 as substrate in culture supernatants in these qualitative assays, unlike Temecula1 or EB92-1/ pSZ26 (PD1703), which clearly did (Fig 2C). Although Temecula1 appeared to exhibit somewhat more lipase activity than EB92-1/pSZ26 (PD1703) (Fig 2C), supernatant protein levels were not quantified, and substrates other than Tween-20 were not investigated.

Hemagglutinin-like proteins have been suggested to be involved in bacterial adherence, aggregation, motility, biofilm formation and virulence of plant pathogens [16–18, 39, 40]. Surprisingly, all six predicted hemagglutinin-like proteins appeared to be missing in EB92-1 [19], even though none of these genes are contiguous in Temecula1 (some are clustered). Two of these (PD1792 and PD2118) carry a type V, two-partner secretion (TPS) domain [41]; the remaining four have no TPS domain. Alignment of these six *X*. *fastidiosa* hemagglutinin like proteins indicated three related clades: one containing both large and small hemagglutinins, including PD0986 (394 aa) and PD1792 (3377 aa); another containing both large and small hemagglutinins, including PD2108 (376 aa) and PD2118 (3457 aa), and a third including PD2110 (436 aa) and PD2116 (438 aa; S4 Fig). In this study, PD0986 (394 aa) was confirmed missing in EB92-1.

Mutations of either large hemagglutinins, PD1792 or PD2118, in Temecula1 caused an increase in virulence and these two genes were identified as anti-virulence genes, causing loss of biofilm formation in *vitro* and in *planta* [16]. Surprisingly, transformation of EB92-1 with the small homolog PD0986 (394 aa), which is most closely related to PD1792 and missing in EB92-1, significantly enhanced pathogenicity of the transformed strain (Figs 4 and 5). The opposite effect might have been expected. The significance of the high degree of similarity of each pair of large and small hemagglutinins, if any, is unknown. It is also not clear how, or even if, the small ones are secreted. The two large hemagglutinins are secreted by TPS Type V secretion system, but the four small ones do not have TPS domains, nor do they appear to be autotransporters, nor do they carry a Type II signal sequence. It is possible that the highly similar ones are self-balancing anti-virulence/virulence pairs.

Zonular occludens toxin (Zot) family proteins are bacterial toxins represented by toxigenic strains of *Vibrio cholera* with the ability to reversibly alter intestinal epithelial tight junctions, allowing the passage of macromolecules through mucosal barriers [42–44]. Zot-like proteins are thought to be of viral origin and are likely to be laterally transferred among microorganisms through the action of filamentous phages [45]. Not surprisingly, the region encoding both Zot genes encodes various predicted phage genes, including PD0911 (replication initiation factor, PD0912 (phage coat protein B), PD0923 (replication initiation factor), PD0933 (phage related protein), and PD0936 (capsid protein). Interestingly, EB92-1 was missing both Zot genes, PD0915 and PD0928, which are identical in sequence, but retained the intervening genes from PD0916 to PD0927 (a total of 11 genes). The fact that both identical proteins in separate locations were missing from Temecula1 may indicate horizontal transfer followed by duplication in a progenitor strain similar to EB92-1, rather than independent deletion events in a Temecula1-like progenitor.

Based on annotation data alone, Zot-like proteins have been predicted and suggested as potential virulence factors in *X*. *fastidiosa* CVC strain 9a5c and other *X*. *fastidiosa* strains, as well as in *X*. *campestris* [45,46]. PD0915 and PD0928 possess a Zot domain (pfam05707) and a ATP-binding cassette (ABC) transporter nucleotide-binding domain (cl21455). ABC transporters are a large family of proteins involved in the Type I transport of a wide variety of different compounds, including sugars, ions, peptides, and more complex organic molecules [47]. pSZ41 (PD0928) conferred increased symptom production to EB92-1, and inoculated grapevines exhibited prominent anthocyanosis with green veinal areas in leaves adjacent to the inoculation zones (Fig 5C and 5D). To our knowledge there has been no previous functional evidence for a role in pathogenicity in *X*. *fastidiosa*, *Xanthomonas*, or any other plant pathogen. The duplication of an identical gene may indicate a selective advantage for *X*. *fastidiosa* strains carrying Zot.

## Supporting Information

S1 Fig PCR results using primers listed in S1 Table to amplify DNA extracted from Temecula1 and EB92-1. Lane 1, Temecula1; Lane 2, EB92-1; Lane 3, water; PCR primer sets (gene target and PCR product sizes) used were: (A) PD1702+3-F and PD1703-R (PD1703, 1.2 Kb); (B) PD0911-F and PD0916-R (PD0915/PD0928, 4.2 kb); (C) ZOT-F and ZOT-R (PD0915/PD0928, 1.4 kb); (D) PD0956-F and PD0956-R (PD0956, 1.0 kb); (E) XFEB114-F and XFEB114-R1 (PD0956, 1.9 kb); (F) PD0956-NF and PD0956-NR (PD0956, 1.5 kb); (G) PD0986-F and PD0986-R (PD0986, 1.2 kb); (H) PD0986-NF and PD0986NR (PD0986, 2.0 kb); (I) RST31 and RST33 (*X*. *fastidiosa* marker gene, 0.7 kb). Molecular weight markers used were Quick-Load 1 kb DNA Ladder from New England Biolabs Inc. (Beverly, MA).
(TIF)

S2 Fig SDS-PAGE gel analysis of protein extracts from induced *E*. *coli* expression cultures used in Fig 3. Lane 1, protein marker (Precision Plus Protein All Blue Prestained Standards, #161–0373, Bio-Rad); Lane 2, total protein from empty vector pET27b cultures ("Total CK"); Lane 3, supernatant protein from empty vector pET27b ("Supernatant CK"); Lane 3, total protein from pSZ37 cultures ("Total LipA"); Lane 4, supernatant protein from pSZ37 cultures ("Supernatant LipA"). Ten μL protein marker and 150–180 ng (5 μL) protein were loaded for analysis. Protein was stained with Coomassie Blue R250. The arrow indicates the expected 42.4 kDa product of PD1703 (LipA).
(TIF)

S3 Fig Dendrogram and multiple sequence alignment generated using PD1702, PD1703, PD1211 and *X*. *oryzae* LipA (LipA). (A). AlignX (Vector NTI Advance 10, Invitrogen, Carlsbad, CA) was used to generate the phylogenetic tree. (B). Residues marked in red are the canonical catalytic catalytic triad residues Ser176, His377, and Asp336 of *X*. *oryzae* LipA. Residues marked in green area are amino acids lining the tunnel with carbohydrate-anchoring pockets.
(TIF)

S4 Fig Dendrogram of 6 predicted hemagglutinin-like proteins from *X*. *fastidiosa* Temecula1. AlignX (Vector NTI Advance 10, Invitrogen, Carlsbad, CA) was used to generate the phylogenetic tree.
(TIF)

S1 Table PCR primers used to attempt detection of selected pathogenicity genes apparently missing in EB92-1. (DOCX)
