# Supplementary figures and images for: Three New Pierce's Disease Pathogenicity Effectors Identified Using Xylella fastidiosa Biocontrol Strain EB92-1
Source: PLoS One. 2015 Jul 28;10(7):e0133796. doi: 10.1371/journal.pone.0133796 (PMC4517913; doi:10.1371/journal.pone.0133796)

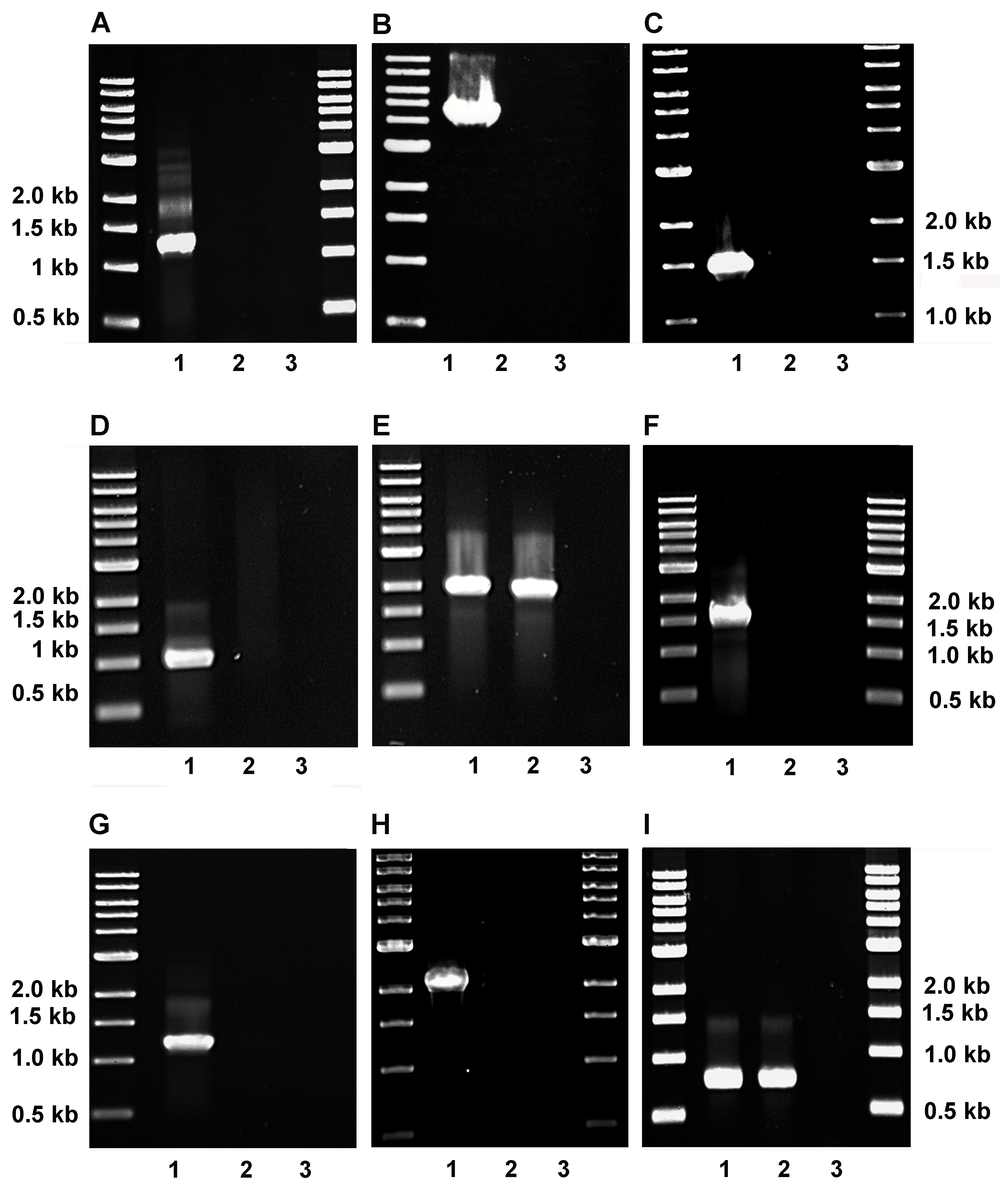

Supplement: S1 Fig — Lane 1, Temecula1; Lane 2, EB92-1; Lane 3, water; PCR primer sets (gene target and PCR product sizes) used were: (A) PD1702+3-F and PD1703-R (PD1703, 1.2 Kb); (B) PD0911-F and PD0916-R (PD0915/PD0928, 4.2 kb); (C) ZOT-F and ZOT-R (PD0915/PD0928, 1.4 kb); (D) PD0956-F and PD0956-R (PD0956, 1.0 kb); (E) XFEB114-F and XFEB114-R1 (PD0956, 1.9 kb); (F) PD0956-NF and PD0956-NR (PD0956, 1.5 kb); (G) PD0986-F and PD0986-R (PD0986, 1.2 kb); (H) PD0986-NF and PD0986NR (PD0986, 2.0 kb); (I) RST31 and RST33 (X. fastidiosa marker gene, 0.7 kb). Molecular weight markers used were Quick-Load 1 kb DNA Ladder from New England Biolabs Inc. (Beverly, MA). (TIF) [file pone.0133796.s001.tif]

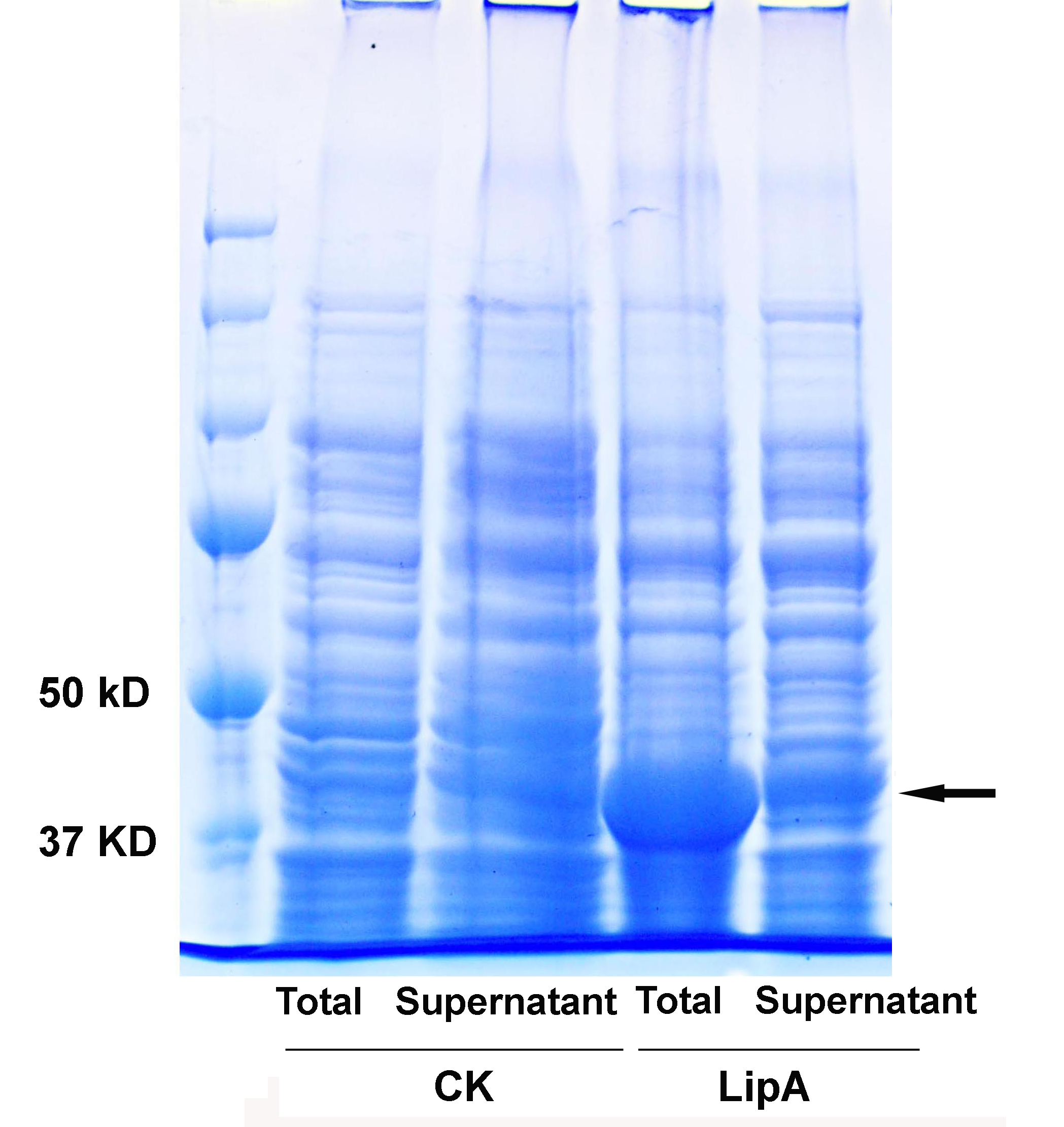

Supplement: S2 Fig — Lane 1, protein marker (Precision Plus Protein All Blue Prestained Standards, #161–0373, Bio-Rad); Lane 2, total protein from empty vector pET27b cultures ("Total CK"); Lane 3, supernatant protein from empty vector pET27b ("Supernatant CK"); Lane 3, total protein from pSZ37 cultures ("Total LipA"); Lane 4, supernatant protein from pSZ37 cultures ("Supernatant LipA"). Ten μL protein marker and 150–180 ng (5 μL) protein were loaded for analysis. Protein was stained with Coomassie Blue R250. The arrow indicates the expected 42.4 kDa product of PD1703 (LipA). (TIF) [file pone.0133796.s002.tif]

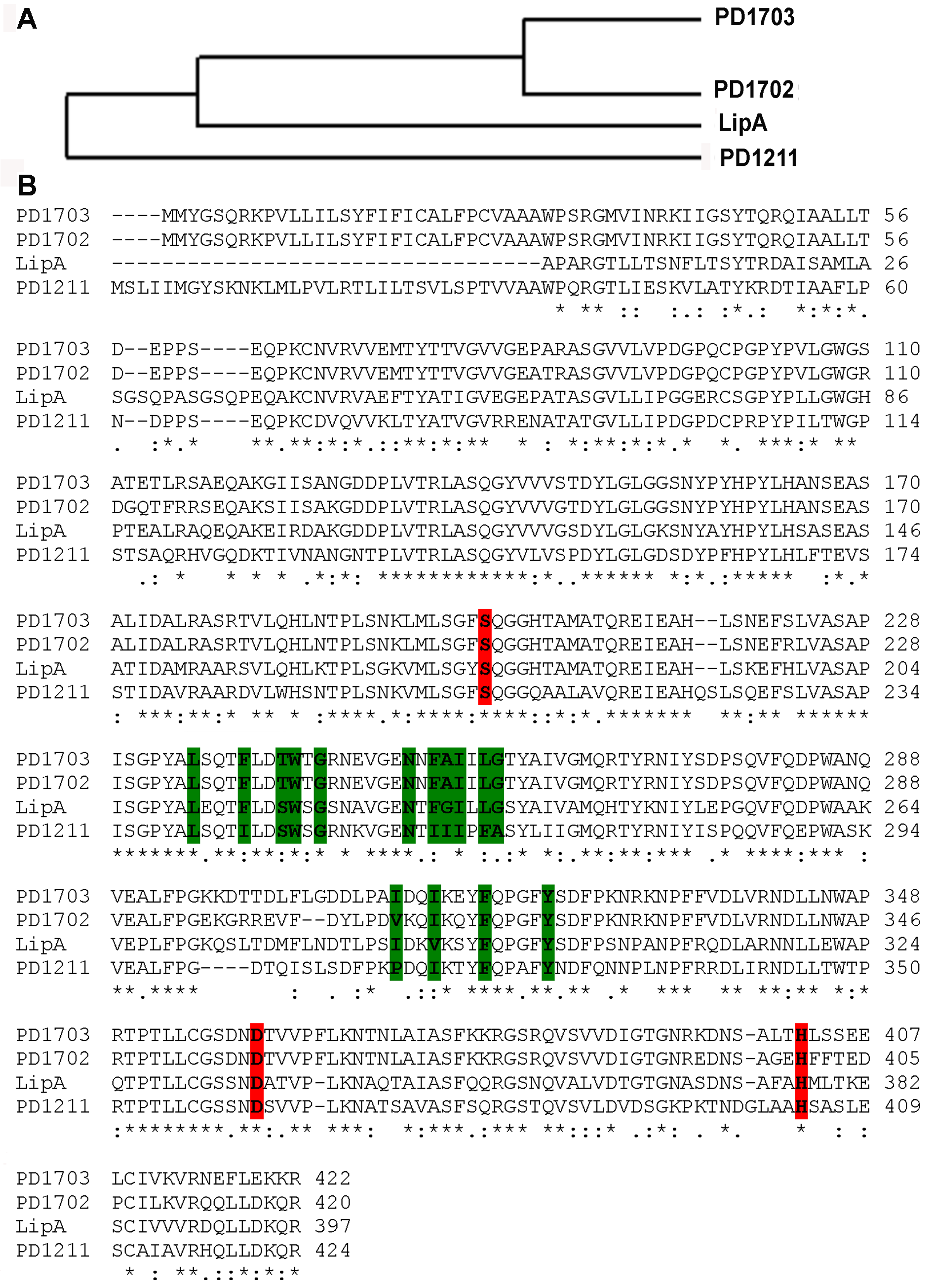

Supplement: S3 Fig — (A). AlignX (Vector NTI Advance 10, Invitrogen, Carlsbad, CA) was used to generate the phylogenetic tree. (B). Residues marked in red are the canonical catalytic catalytic triad residues Ser176, His377, and Asp336 of X. oryzae LipA. Residues marked in green area are amino acids lining the tunnel with carbohydrate-anchoring pockets. (TIF) [file pone.0133796.s003.tif]

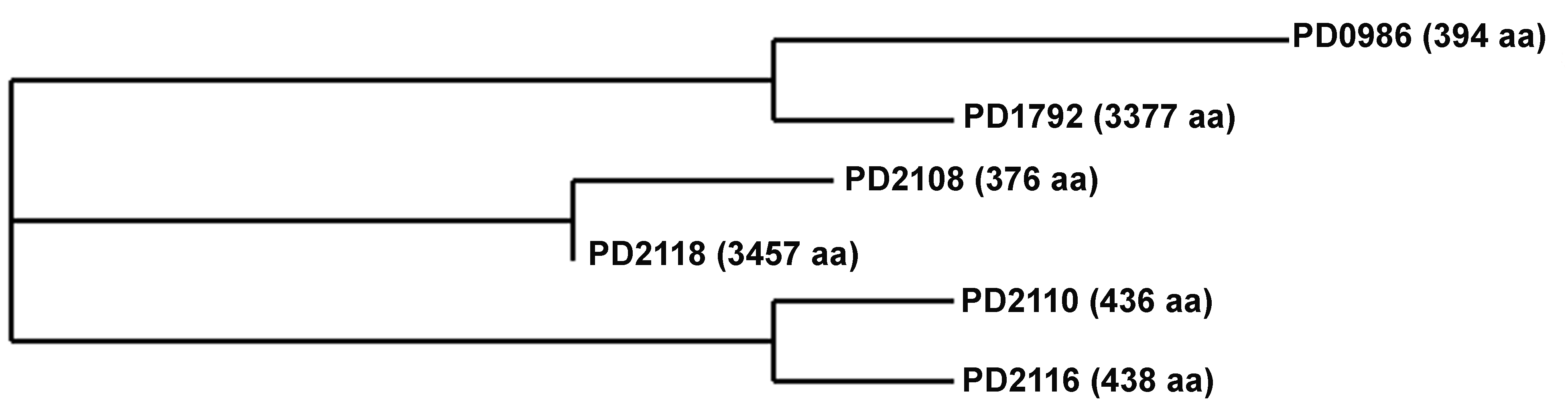

Supplement: S4 Fig — AlignX (Vector NTI Advance 10, Invitrogen, Carlsbad, CA) was used to generate the phylogenetic tree. (TIF) [file pone.0133796.s004.tif]
