# Supplementary material for: Three New Pierce's Disease Pathogenicity Effectors Identified Using Xylella fastidiosa Biocontrol Strain EB92-1
Source: PLoS One. 2015 Jul 28;10(7):e0133796. doi: 10.1371/journal.pone.0133796 (PMC4517913; doi:10.1371/journal.pone.0133796)
Supplement: S1 Table — (DOCX) [file pone.0133796.s005.docx]

- **Table S1. PCR primers used to attempt detection of selected pathogenicity genes apparently missing in EB92-1.**

| - **Primer name** | - **Sequence (5’-3’)** | - **Primer location^a^** | - **EB92-1 Contig** | - **Gene target** |
| --- | --- | --- | --- | --- |
| - PD1702+3-F | - GTGATTAACAGAAAAATTATTGGT | - Outside | - Contig00115 | - PD1703 |
| - PD1703-R | ACGTTTCTTCTCTAGAAACTCA | - Outside | - Missing |  |
| - PDLPwp-F | - GGGCCCTGCCGCATTTGAGGCTGGC | - Outside | - Contig00115 |  |
| - PDLP-R | - ATCGATAGGGTACATTTACCAGACCG | - Outside | - Contig00115 |  |
| - PD0956-NF | TCAGCGACAT TGATCCATCG | - Outside | - Missing | - PD0956 |
| - PD0956-NR | TTATCTCCCAAAATCATCA CC | - Outside | - Missing |  |
| - XFEB114-F | TGAGGCTGCTCATCTTGCTA | - Outside | - Contig00114 |  |
| - XFEB114-R1 | GCATTCGCAGTACAAAACGA | - Outside | - Contig00114 |  |
| - PD0911-F | - TTGAAGTTGGGGACTGATCC | - Outside | - Missing | - PD0915/PD0928 |
| - PD0916-R | - CCACACACACCCAAGAAATG | - Outside | - Missing |  |
| - ZOT-F | - GTCGACCTGGTTGGGCATTAATTGGG | - CDS | - Missing |  |
| - ZOT-R | - GGATCCTCATGGAAGCGACCCCGC | - CDS | - Missing |  |
| - PD0986-F | - TCACTCAAGTCATGAACCGC | - CDS | - Missing | - PD0986 |
| - PD0986-R | - TTAGTAGGGGGTGAGGGAC | - CDS | - Missing |  |
| - PD0986-NF | - GTCTTGCTCCCTGCTGGCCG | - Outside | - Missing |  |
| - PD0986-NR | - GGATGCAGGGACATAAGGG | - Outside | - Missing |  |
| - RST31 | - GCGTTAATTTTCGAAGTGATTCGATTGC | - / | - / | - *X. fastidiosa* marker gene [27] |
| - RST33 | - CACCATTCGTATCCCGGTG | - / | - / |  |

- ^a^ Location in Temecula1, either inside the CDS or Outside of the CDS
